# Well-Being, Depression, and Anxiety following Oncoplastic Breast Conserving Surgery versus Modified Radical Mastectomy Followed by Late Breast Reconstruction

**DOI:** 10.3390/ijerph18179320

**Published:** 2021-09-03

**Authors:** Daciana Grujic, Cătălina Giurgi-Oncu, Cristina Oprean, Zorin Crăiniceanu, Ica Secoșan, Ioana Riviș, Ion Papavă, Corina Vernic, Cristina Bredicean

**Affiliations:** 1 Department of Plastic and Reconstructive Surgery, “Victor Babeș” University of Medicine and Pharmacy, 300041 Timișoara, Romania; dcalistru@yahoo.com (D.G.); zcrainiceanu@gmail.com (Z.C.); icasecosan@yahoo.com (I.S.); 2 Department of Plastic and Reconstructive Surgery, “Pius Branzeu” Emergency Clinical Hospital, 300723 Timișoara, Romania; 3 Discipline of Psychiatry, Department of Neuroscience, “Victor Babeș” University of Medicine and Pharmacy, 300041 Timișoara, Romania; papava.ion@umft.ro (I.P.); bredicean.ana@umft.ro (C.B.); 4 “Eduard Pamfil” Psychiatry Clinic, “Pius Branzeu” Emergency Clinical Hospital, 300425 Timișoara, Romania; 5 Discipline of Morpho-Pathology, “Victor Babeș” University of Medicine and Pharmacy, 300041 Timișoara, Romania; coprean@yahoo.com; 6 Department of Oncology, ONCOHELP Hospital, 300239 Timișoara, Romania; 7 Department of Oncology, ONCOMED Outpatient Unit, 300239 Timișoara, Romania; 8 Department of Neurosciences, “Carol Davila” University of Medicine and Pharmacy, 020021 Bucharest, Romania; ioana.rivis@drd.umfcd.ro; 9 Discipline of Medical Informatics and Biostatistics, Department of Functional Sciences, “Victor Babeș” University of Medicine and Pharmacy, 300041 Timișoara, Romania; cvernic@yahoo.com; 10 Psychiatry Inpatient Unit, Internal Medicine Clinic, “Dr. Victor Popescu” Military Emergency Clinical Hospital, 300080 Timișoara, Romania

**Keywords:** breast cancer, conservative surgery, anxiety, depression, well-being

## Abstract

Breast cancer requires complex clinical care. Well-being is an intricate concept, encompassing physical, functional, emotional, and social aspects. Background: This study aims to evaluate the relationship between the type of surgery our patients underwent and the timing of the reconstructive surgery with physical, emotional, social, and functional well-being. Furthermore, through our research we tried to identify potential mental health comorbidities in patients with breast cancer, clinical symptoms, and well-being in women with breast cancer, depending on the type of required surgery. Methods: The study included 69 women diagnosed with breast cancer, in stages I to III, divided in two groups: I—patients with oncoplastic breast-conserving surgery and contralateral correction surgery, for symmetry reasons; II—patients who underwent modified radical mastectomy and late breast reconstruction with contralateral symmetrisation. We evaluated socio-demographic aspects, alongside depression, anxiety, stress (DASS 21), and well-being (FACT-B). Data were statistically processed; statistical significance was set at *p* < 0.05. Results: Clinical elements of depression, anxiety, and stress were noted in both groups, without statistical significance (*p* > 0.05). Significant differences were found regarding psycho-emotional (*p* = 0.035) and functional well-being (*p* = 0.001), with higher scores for group I. The chi-square test indicated statistically significant differences (at *p* < 0.01) between the groups, regarding the frequency of scores on items B4 and B9 (FACT-B items, related to feminine aesthetics and desirability), with evidently higher scores in group I than in group II. Conclusions: The state of well-being, as well as the items related to femininity and sexuality had higher values in the group of women treated by oncoplastic conservative surgery compared to late reconstruction after modified radical mastectomy.

## 1. Introduction

Quality care for a person with breast cancer includes dignity, respect, support, and kindness, involving not only the somatic impact of the disease, but also the social, emotional, and intimate well-being. Maintaining one’s dignity, with respect for individual needs and preferences, is one of the goals we take into account when deciding upon the most appropriate therapeutic plan. It can be a lengthy process, throughout disease evolution, until the person with breast cancer regains their purpose and life dignity. This approach means that we must allow the person with breast cancer to take part in decisions, but also to help them achieve balance on all levels—physical, emotional, spiritual, and social [1]. In Romania, according to estimates in the GLOBOCAN 2018 status report on the global burden of cancer worldwide, in a female population of 10,095,020, the total number of neoplasm cases was 38,439. Of these cases, 9629 (25.1%) women were diagnosed with breast cancer [2]. Well-being is an intricate concept, tangential to other terms, such as quality of life, life satisfaction, and psychological well-being; it requires identification of what one thinks and feels about one’s own life, such as the quality of relationships, positive emotions and resilience, the realization of one’s potential, or one’s overall satisfaction with life—i.e., the subjective “well-being”. Well-being generally includes global judgments on life satisfaction, as well as feelings, ranging from depression to joy, and people’s beliefs, in terms of their lives, their own evaluation of the quality of their relationships, emotions, or their ability to adapt. It is for these reasons that subjective and psychological well-being, as well as quality of life, may sometimes be equivalent. According to the World Health Organization, subjective well-being (correlated with mental health) consists of three aspects: Emotional, psychological, and social. 

Psychological well-being includes two components: Global judgments regarding life satisfaction (the cognitive component) and feelings about life experiences, and the roles a person holds (the affective component). Both components are essential, as they contribute to the achievement of adequate social functioning [3].

Regarding social aspects, in order to resolve any interpersonal conflicts and, subsequently, to achieve well-being, we require skills that allow us to infer attitudes about the mental states of others (theory of mind). Theory of mind abilities may be assessed by using several instruments, such as the “Reading the Mind in the Eyes” test, the short story task, visuospatial skills testing, or humor comprehension [4].

A diagnosis of breast cancer generates profound changes in a woman’s life, mainly caused by the inherent vital risk, but also due to the meaning of breasts for one’s self-image. The breasts harmonize in the mind and life of a woman with aspects of femininity, sexuality, and fertility. The entire therapeutic plan that follows the diagnosis of breast cancer gives way to a certain level of stress, since this diagnosis is associated with a series of physical as well as mental challenges related to body appearance, in conjunction with a succession of associated future experiences, and possibly death. Like any neoplastic condition, the treatment is a complex and individualized one, comprising repeated surgeries, associated with individualized oncological treatment: Chemotherapy, radiotherapy, as well as hormone therapy in certain cases. Although each of these therapeutic interventions has its benefits, there also many, potentially severe, side effects.

In general, the excision of part of or the entire breast can leave visible scars, deformities, or major asymmetries of the breast region, with the psychological impact of these changes on the body possibly reflected in the development of poor mental health, such as anxiety, depression, body-image disorders, or a variety of social dysfunctions, including sexual intimacy difficulties [5,6]. It is worth remembering that there are (few) women in whom surgery does not affect the way they subsequently feel or function, but most women find these changes difficult to accept. They may feel that they are losing their femininity, that they have become unattractive, they may suffer from reduced self-confidence, even from low self-esteem—all of which can be detrimental to their daily lives [7]. 

Although many of the physical effects caused by breast cancer treatment may be temporary, especially those that occur following chemotherapy (such as hair loss, dentition damage), there are some effects that become permanent and troublesome, such as breast deformity, associated with breast asymmetry. Besides, there are much more serious and even life-threatening side effects, determined by radiotherapy treatment (pericarditis, pulmonary fibrosis, fibrosis of the coronary vessels at risk of thrombosis, or others).

The surgical treatment of breast cancer has made great strides over the years, with the transition from aggressive and mutilating surgeries to conservative surgical treatments, associated with oncological treatment: Radiotherapy, chemotherapy, and hormone therapy.

Since the 1990s, the concept of oncoplastic surgery has been introduced precisely to improve the aesthetic and functional results of the breast [8,9,10]. This type of surgery is a multidisciplinary collaboration that combines oncological surgery with plastic surgery techniques, thus offering women diagnosed with breast cancer a better quality of life in the long run [11,12,13]. 

The type of surgery is chosen depending on the tumor biology, size, localization, and number of tumors in the breast. The ratio of the tumor/breast volume also represents an important factor in choosing the type of surgery. The most appropriate individual mode of surgical treatment is decided by the multidisciplinary team and presented to the patient. The patient will/will not agree with the proposed treatment and will take note of the stage of treatment by discussing each aspect with the medical team.

In the literature, several studies have presented results reported by patients on their physical and psychosocial well-being, which was superior when oncoplastic surgery techniques were applied, compared to conventional surgery [14,15]. Several studies reported that oncoplastic surgery has a positive result, in terms of women’s quality of life, as well as superior aesthetic results [16,17].

Breast reconstruction is the procedure that restores the shape and size of the breast that was lost in the mastectomy operation. This can ensue in one or several stages, immediately after the modified radical mastectomy (in the same operation) or later, one year or more after the mastectomy. Immediate breast reconstruction helps maintain quality of life following surgery, allowing the patient to preserve an adequate body image and a positive attitude. However, about half of women do not opt for breast reconstruction [18]. The reasons why so many patients refuse this medical procedure remain largely unclear. In addition to certain demographic characteristics, the stage of the tumor, and the incomplete or substandard information provided by the attending physician, there are data in the literature that suggest that the psychological profile of the woman may play a decisive role in their decision regarding the reconstruction procedure [19].

As is the case globally, in Romania, breast cancer represents a critical pathology, both epidemiologically and socially; since 2014, this reality led to the development of a national program for breast reconstruction following oncological diseases, entirely financially supported by the government. However, the number of people who have chosen to benefit from this program is minimal (5%), even though, internationally, there has been an increase in the rate of both immediate and delayed breast reconstruction in recent decades [20,21]. The feeling of worry or sadness that women diagnosed with breast cancer carry in their daily lives can remain for years after diagnosis and treatment [22,23]. In addition to psychiatric symptoms, there are also physical disturbances, such as vasomotor symptoms, lymphedema/arm discomfort, atrophic vaginitis, pain, nausea, and emesis, which may be direct consequences of the pathology, or treatment side effects. All of these aspects tend to negatively influence one’s well-being. 

This paper aims to evaluate the relationship between the type and timing of the reconstructive surgery with the physical, emotional, social, and functional well-being, and to identify potential mental health comorbidities in patients with breast cancer.

## 2. Materials and Methods

Study group: This study is a cross-sectional analysis evaluating a small group of women diagnosed with breast cancer, who underwent surgical treatment by either conservative oncoplastic breast-conserving surgery, or modified radical mastectomy followed by late reconstruction, from July to December 2019 in the Plastic Surgery Department of the Timișoara County Emergency Clinical Hospital in Romania. Of 108 hospitalized women, only 69 (63.8%) met the inclusion criteria and were, therefore, admitted in the study.

The study was approved by the Local Scientific Research Ethics Committee of the Timişoara County Emergency Clinical Hospital. Informed consent was obtained from all individual participants included in the study. Due to the small number of subjects, we based the selection on inclusion/exclusion criteria, without the use of statistical methods. It should be noted that the study samples are statistically comparable in terms of age and marital status. 

### 2.1. Patient Selection

#### 2.1.1. Inclusion Criteria

- The first admission in 2018–2019 with a diagnosis of breast cancer, according to the WHO ICD-10 criteria [24].
- Women aged over 18 years.
- The stage of the neoplasm considered the international classification: I, II, IIIA, III B.
- Patients who underwent either oncoplastic breast-conserving surgery or late reconstruction after a modified radical mastectomy.
- All patients were evaluated between 3 and 6 months after the last surgical intervention.
- All subjects agreed to participate in the study.

#### 2.1.2. Exclusion Criteria

- Presence of learning difficulties (IQ < 90)
- Presence of a mental illness caused by psychoactive drugs or an organic disorder.
- Patients who underwent radical modified mastectomy and refused subsequent reconstructive surgery.
- Patients with severe comorbidities where the reconstructive surgery was contraindicated.
- Patients diagnosed with breast cancer stage IIIC, metastatic or recurrent disease.

The study was performed on 69 patients, with various breast neoplasm staging, namely I, II, IIIA, and IIIB. Patients were then divided into 2 groups, according to the type and timing of the agreed-upon surgical reconstruction. In group I we included 34 patients, who underwent oncoplastic conservative surgery +/− sentinel lymph node excision/total lymphadenectomy. Group II comprised 35 patients who underwent modified radical mastectomy, performed in a General surgery/Oncology Surgery Department. The breast reconstruction was initiated at a later date, a minimum of one year after the primary surgery or immediately after finalizing the chemotherapeutic treatment.

The most common histological subtype of breast cancer in patients included in group I was the infiltrating ductal carcinoma, 32 cases (94.11%), while 5.88% were diagnosed with lobular carcinoma. A percentage of 61.76 (21 patients) had stage I, 38.23% (13 patients) were diagnosed with stage II, and 14.70% (5 patients) were stage III according to TNM stage distribution AJCC 8th edition. As for the subtypes, 85.29% (29 patients) were diagnosed with Luminal A or B breast cancer and 14.71% (5 patients) were HER2 positive. There were no TNBC (triple negative breast cancer) patients included in this study and 35.29% of the patients in group I received neoadjuvant chemotherapy.

The type of intervention was:

- Enlarged tumorectomy with tumor-free resection margins, followed by reconstruction of the remaining defect by a minor mobilization of the remaining glandular tissue, with/without NAC excision, with/without NAC positioning.
- Enlarged sectorectomies and immediate reconstruction using mammoreduction techniques adapted to the initial tumor positioning, associated with contralateral mammoreduction for symmetrization
- The remaining defect after tumor excision was filled with muscle/musculocutaneous flaps of latissimus dorsi muscles and perforator-based flaps. For this group, symmetrization of the contralateral breast (mostly mammoreduction) is performed in the same surgical time.

All patients in group I underwent adjuvant radiotherapy treatment, 44.11% chemotherapeutic treatment, and 14.70% anti-HER2/neu therapy. Every patient diagnosed with the Luminal subtype received adjuvant hormonal-therapy.

In group II, we included 35 patients, 20% of whom had stage I, 37.14% stage II, and 42.85% stage III breast cancer. Regarding the histological subtype, 81% had infiltrative ductal carcinoma. As for the subtype, 77.14% had Luminal A or B and 15% were HER2 positive. A total of 8 patients in group II (22.85%) were diagnosed with triple negative breast cancer. A majority of 80% of the patients received neoadjuvant chemotherapy +/− anti HER2 therapy, while 74.28% received adjuvant radiotherapy. Patients with the Luminal subtype received hormonal-therapy. 

The late reconstruction was performed either in a single surgical time, opting for a Becker implant or reconstruction with autologous tissues (latissimus dorsi, right abdominal muscles, gluteus, or microsurgical flaps on DIEP perforators), or in two stages, namely with an expander and an implant. It should be mentioned for this group that the symmetrization of the contralateral breast was practiced 6 months after the stabilization of the reconstructed breast. It is imperative to note that, routinely, for all subjects hospitalized in this Department, the onsite clinical psychologist performs a complex mental health evaluation, including a Mini Mental State Examination (MMSE) test, and determines the IQ. Thus, an initial, preoperative, mental health evaluation was performed for all patients included in this study, which did not reveal concerns with regards to clinical symptoms of anxiety or depression. None of the patients reported being under psychological or psychotropic treatment for a mental illness at the time of inclusion in this study. Routine biological samples and screening tests for psychoactive substance misuse were also performed.

### 2.2. Evaluation

We used the following clinical instruments: A survey with general demographic questions, the FACT-B, and the DASS-21R Scale. Both scales are validated for the Romanian population. The socio-demographic questionnaire included data regarding age, educational level, marital status, and background of subjects. 

After selecting the study group, the socio-demographic questionnaire, as well as the two assessment scales were applied in the same session. 

The state of well-being was assessed with the Romanian language version of the FACT-B instrument. The questionnaire contains 37 items, grouped into 5 subscales, scored on a 5-point Likert scale, where 0 means “not at all” and 4 means “a lot”. Measured items include Physical Well-being (7-item), Social/Family Well-being (7-item), Emotional Well-being (6-item), Functional Well-being (7-item), and the Additional concerns/Breast Cancer Subscale (10-item). The scores for each subscale are 0–28 for Physical Well-being, 0–28 for Social/Family Well-being, 0–24 for Emotional Well-being, and 0–28 for Functional Well-being. Higher scores correlate with the presence of a state of well-being. For the purposes of this study, we chose to highlight two specific items included in the Additional concerns/Breast Cancer Subscale, namely B4 (“I feel sexually attractive”) and B9 (“I am able to feel like a woman”), as they directly relate to the concept of feminine aesthetics and sexuality, representing some of the more immediate and serious concerns of women living with the consequences of breast cancer surgery [25].

The DASS-21, a self-report questionnaire consisting of 21 items, has 7 items per subscale: Depression (Cronbach’s alpha for this scale was α = 0.88), anxiety, and stress (Cronbach’s alpha for this scale was α = 0.91). Participants were asked to score every item on a scale from 0 (did not apply to me at all) to 3 (applied to me very much). The scores have the following interpretations: Depression (normal 0–4, mild 5–6, moderate 7–10, severe 11–13, extremely severe over 14), anxiety (normal 0–3, mild 4–5, moderate 6–7, severe 8–9, extremely severe over 10), and stress (normal 0–7, mild 8–9, moderate 10–12, severe 13–16 extremely severe over 17) [26].

### 2.3. Statistical Data Processing

Statistical analyses were conducted with Stata 16.1 (Statacorp, College Station, TX, USA). Descriptive statistics (mean, standard deviation, percentage) were calculated. Student’s *t*-test was used to compare mean values between the reconstructive surgery group and initial mastectomy, and the subsequent breast reconstruction group. Chi-squared tests were used to evaluate the differences between the two groups with respect to different characteristics. Given that the sampling was performed on the basis of clinical inclusion/exclusion criteria, the sample was not statistically representative. A probability level of *p* < 0.05 was considered to indicate statistical significance.

## 3. Results

The study included a total of 69 women, divided into two groups: Group I, 34 women, who underwent oncoplastic breast-conserving surgery and contralateral symmetrization; and group II—35 women—who underwent a modified radical mastectomy, followed by delayed reconstruction and symmetrization. 

The average age of subjects included in group I was 49 years (std. dev. = 8.9 years), with a minimum age of 29 years and a maximum age of 67 years; the mean age of subjects in group II was of 57.9 years (std. dev. = 7.1 years), with a minimum age of 32 years and a maximum age of 67 years. The difference in averages was statistically significant (*p* < 0.001), which may be explained by the fact that, generally, younger people tend to have and invest more time and care in their appearance.

Table 1 summarizes the collected demographic data, comparatively, for the two groups. There are no statistically significant differences between groups, in terms of the frequency of the presence in any of the analyzed socio-demographic categories, indicating that none of the socio-demographic factors that were explored (marital status, educational status) could be associated with personal choice, in terms of the type of performed surgery.

The two groups were relatively homogeneous, and most patients reported a high-school education, a married status, and an urban residence. 

We compared the frequency of the presence of various levels of depression, anxiety, and stress in the two groups. To determine the level of depression, anxiety, and stress, we used the DASS-21 scale, which comprises 3 distinct subscales. Table 2 shows the distribution of subjects into the two groups, depending on the intensity of depressive, anxiety, and stress symptoms, according to the DASS-21 Scale.

Student’s t test was used to analyze the differences between the mean scores of the three sub-scales for the two groups. The results are shown in Table 3.

The DASS-21 questionnaire was employed to identify the levels of depression, anxiety, and stress; its results showed some differences between the two groups (although, not statistically significant).

Regarding the depressive features, these were reported by more than 70% of all patients, albeit, more frequently by women in group II (however, without any statistically significant differences (*p* = 0.812). Anxiety was also present in over 70% of all women included in this study, with a slightly higher frequency in group I (however, without suggesting statistically significant differences (*p* = 0.985). Stress was noted in approximately 25% of the entire study sample, and at a somewhat higher rate in group I (however, without indicating statistically significant differences (*p* = 0.383). For the (self-evaluation) analysis of the well-being of subjects, we used the FACT-B instrument, which evaluated the four components: Physical, social, psycho-emotional, and functional status. Table 4 compares the averages of scores obtained by the subjects from the two groups on the items of this test. Student’s t test was used to analyze the differences between the averages.

We found statistically significant differences between the two groups, in terms of the average scores obtained on two specific items of the FACT-B test: Psycho-emotional well-being and functional well-being. In both cases, the subjects in group I obtained better scores than those in group II, indicating that women who underwent oncoplastic breast-conserving surgery interventions and contralateral symmetrisation subsequently had a better psycho-emotional and functional well-being. Higher scores were also obtained by subjects in group I on the specific items regarding social well-being, but the differences between groups were not statistically significant.

We also compared the frequencies for the two specific items related to feminine aesthetics and sexuality (B4 and B9), as presented in the tables below (Table 5 and Table 6).

The chi-square test indicated statistically significant differences between the groups, in terms of the frequency of scores for the two FACT-B items that refer to femininity (*p* = 0.001) and desirability (*p* < 0.001). Accordingly, it can be noted that almost twice as many subjects from group I (85.2%) obtained high scores (3 and 4) on item B9 (“I am able to feel like a woman”), compared to subjects from group II (42.8%); likewise, 94.1% of the subjects in group I obtained high scores (3 and 4) on item B4 (“I feel sexually attractive”), compared to naught (0.0%) subjects in group II.

## 4. Discussion

Breasts are one of the main symbols of femininity. The psychological and social impact of their absence or deformity began to be highlighted at the time of implementation of surgical treatment in breast cancer [27,28]. For decades, in women diagnosed with breast cancer, the emphasis of the therapeutic conduct has been on oncological safety, leaving aside the aesthetic result, or the evaluation of individual well-being. Understanding the life-altering impact that a mastectomy surgery may have on a woman’s psychology should be a key principle for medical professionals, allowing them to be better equipped when informing the patient about the changes caused by the procedure. Subsequently, any decisions made must be mutually agreed, in order to ensure the physical and mental well-being of the patient.

Clarification regarding physical changes, as well as on the perception of one’s own body, the interference with oncological safety, and individual quality of life are fundamental concepts that must be adhered to, in order for professionals to be able to elaborate a successful treatment plan, when dealing with breast cancer. Recently, there has been increased interest in the psychological phases that may occur in a woman diagnosed with breast cancer, most notably, in the emotional problems represented by depression and anxiety, in addition to the oncological treatment, which, in itself, may cause other physical and mental disorders. In this study, we tried to answer some questions that arise regarding the relationship between the type of surgery (classic or conservative), well-being, and the presence of depressive and anxious elements in women with breast cancer. 

The two groups that we evaluated in this study were relatively homogeneous in terms of socio-demographic data, except for the average age of patients, which was found to be younger in group I. The presence of clinical symptoms of depression, anxiety, and stress was identified by using the DASS-21 self-assessment scale, which performs an assessment based on size, rather than on the categorical concept. The presence of depressive elements was evident, postoperatively, in about three-quarters of women in group I and group II. Comparing the two groups, we noticed that women in group II (with modified radical mastectomy and late reconstruction) reported more depressive features, but without any statistically significant differences. It should be mentioned that the evaluation was performed 3–6 months following the final surgery.

Anxiety was another mental ill health complaint reported by most patients in this study; this psychopathological feature was found to be more frequent in group I, without statistically significant differences evident between the two groups. Given that women in group II have undergone previous surgery, it might be plausible that their anxiety levels were lower at the time of our evaluation. Stress, in contrast, was reported only by a small number of women, without statistically significant differences between the two groups.

In terms of well-being, the FACT-B test evaluated the four components (physical, emotional, social, and functional well-being), and included a specific subscale for particular aspects of breast cancer, consisting of 10 items. For the purposes of this paper, results regarding two of the FACT-B Additional concerns/Breast cancer subscale items (B4 and B9) are discussed in more detail (even though, all items were duly evaluated). We chose to focus on these items (B4—“I feel sexually attractive” and B9—“I am able to feel like a woman”), as they are the most representative when it comes to aspects related to femininity, aesthetics, desirability, and intimacy.

In all four components of well-being, the averages of the acquired scores were high, which shows that there is a certain degree of physical, social, psycho-emotional, and functional well-being in both groups. The group of women treated by oncoplastic conservative surgery attained higher scores on all well-being components, compared to the group that underwent a mastectomy and late reconstruction.

What differentiated the two groups most were the aspects of emotional and functional well-being, with statistically significant differences. Group I, which included women who underwent oncoplastic breast-conserving surgery, reported statistically significant higher emotional well-being compared to group II. The concept of emotional well-being assesses the perspective of depression, resilience to illness, hope, anxiety, but also fear of death, all of which constitute essential components when discussing a person’s emotional nature. The impact of this particular aspect of well-being affects quality of life for this patient category. The comparison of the emotional well-being of women treated by conservative surgery with that of the other group, who chose classical surgery followed by subsequent reconstruction, highlights the central role this procedure plays when it comes to one’s affect.

The literature describes several studies that evaluated the relationship between surgery, well-being, and quality of life in women diagnosed with breast cancer. A study by Dean and Crittenden assessed quality of life in women who received breast reconstruction, preoperatively, and at 6 months postoperatively, observing an increase in the psychosocial, physical, and sexual well-being, when comparing preoperative with postoperative scores [29]. In both of our study groups, the mental health evaluation performed preoperatively failed to highlight clinical levels of anxiety or depression. This may, in part, be explained by the cultural reticence of Romanians to acknowledge/disclose mental health difficulties due to fear of social stigmatization or fear of oncological treatment delay. 

Another study conducted by Susarla et al. aimed to assess the outcomes of immediate, single-stage, implant-based reconstruction, compared with traditional, two-stage reconstruction (i.e., tissue expander placement followed by an exchange to implant). The study results suggested there was no significant difference in complication rates between single-stage versus two-stage implant-based breast reconstructions. Although single-stage reconstruction is associated with higher sexual well-being satisfaction, it is also more than 80 percent more likely to require additional operative revisions. Two-stage reconstruction is associated with significantly higher satisfaction with the medical and care team [30]. Additional research conducted by Ng SK et al. confirmed the positive effects of breast reconstruction post-mastectomy and identified the reasons that influence patients’ decisions of whether to undergo reconstruction. Breast reconstruction should be seen as an integral part of the comprehensive care of women with breast cancer and an important health care priority [31]. 

The evaluation of the two specific FACT-B items, B4 (“I feel sexually attractive”) and B9 (“I am able to feel like a woman”), showed that there were statistically significant differences between the two groups, with over 80% of women in group I reporting significant results, i.e., scores of 3–4 (“quite a bit” and “very much”), compared to group II. Breast reconstruction aims to restore body image, while minimizing the changes caused by surgery and, therefore, improving the overall quality of life [32,33]. Essentially, the two items explore the two aspects where breasts play important roles: That of femininity, but also in one’s intimate life and relationship satisfaction. The concept of femininity is defined as a set of attributes, behaviors, and roles that are largely a social construct, located at the intersection of social and biological factors. On the one hand, there is the issue of a woman’s relationship with her own body and, on the other, that of her place in a couple and in the wider society. For each woman, this concept has a different value system; feeling feminine and sexually attractive may be just as important as having a fulfilling intimate life and, implicitly, a satisfactory relationship.

In the literature, there are clinical studies on the sexual satisfaction of women with surgically treated breast cancer. Howes et al. showed that women who benefited from immediate breast reconstruction had better indicators, in terms of sexual satisfaction, compared to those who had a total mastectomy without reconstruction. The latter group subsequently presented deformities of the breast and asymmetries that led to a decrease in their self-esteem [16]. This serious change in one’s own body image is often associated with depressive symptoms, as well as with significant levels of dissatisfaction with social and sexual issues, preventing the person from maintaining an adequate quality of life [17,34]. Research led by Shekhawat et al. revealed that breast reconstruction had a positive impact on the satisfaction regarding the physical appearance of breasts, as well as on the psychosocial, sexual, and physical individual well-being. They also showed that women who did not benefit from breast reconstruction were faced with intimacy problems [17]. Similarly, it was observed that the degrees of depression, anxiety, and stress correlate statistically significantly inversely with functional well-being in women treated with conservative surgery, while in the group treated by mastectomy followed by reconstruction, it correlates with emotional and physical well-being. 

Functional well-being appreciates the ability to work, to enjoy life, to accept illness, as well as the level of individual quality of life and sleep; emotional well-being appreciates sadness, irritability, worry, loss of spontaneity, while physical well-being evaluates energy, pain, and time spent in bed. Women treated by mastectomy followed by late reconstruction initially underwent the experience of a mutilating surgery, having to spend a significant period of time living with visible physical changes; as a result, their emotional and physical lives were negatively influenced. The same issues were not found in women who underwent conservative surgery, even though they reported depressive, anxiety, and panic features. The presence of depression, anxiety, and stress are also inversely correlated with the two specific items, B4 (“I feel sexually attractive”) and B9 (“I am able to feel like a woman”), in both groups. Regarding item B4, which assesses how attractive a woman feels, it was observed that this is influenced by depression, anxiety, and stress, alike, in both groups. It is interesting to note that the score on item B9, which expresses the concept of femininity, seems to be influenced in group I by stress level, while in group II, this was influenced at a higher degree by the levels of anxiety and depression. The concept of femininity contributes to the achievement of the level of well-being that every woman treated for breast cancer should, ideally achieve postoperatively. Although there are clinical elements of depression, anxiety, and stress in both groups, alongside an inverse correlation between these elements and items B4 and B9, women who benefited from conservative surgery showed much higher scores on these items, which express desirability and femininity, compared to the other group.

Since 2014, the Romanian government has been funding a national program for breast reconstruction by endoprosthesis following oncological illnesses; this saw the achievement of many more cases of immediate breast reconstruction than in previous years, but we are still very far from the percentages that exist worldwide for this type of surgery. Globally, 60–80% of patients, who are treated by mastectomy, benefit from reconstruction. However, until 2016, in our country, there were only 176 women who benefited from this procedure, even though the number of cases diagnosed yearly in Romania is of several thousands; for example, in 2017, there were 7499 cases [34].

In recent years, the clinical standard involves the discussion of each individual breast cancer case within the Multidisciplinary Team, so that patients benefit from the experience and proficiency of several doctors: The oncologist considers surgical approaches, the plastic surgeon examines the reconstruction procedure, the pathologist reviews the characteristics and changes of the examined tissues, the imaging doctor presents aspects related to tumor staging, the oncologist discusses the approaches in chemotherapy and radiotherapy, while the psychiatrist evaluates the presence of clinical elements suggesting mental health and advises on best practices towards obtaining well-being [35].

Regarding the limitations of this research, we highlight the small number of cases, which might have had an impact on the conclusions that resulted. 

We believe that the results could have shown greater differences in terms of well-being if we had compared women who benefited from oncoplastic conservative surgery with women who only benefited from modified radical mastectomy. Additionally, we used self-assessment scales, both pre- and postoperatively, which are instruments that may be influenced by individual levels of education, the given clinical situation, as well as by individual personality traits.

## 5. Conclusions

The multidisciplinary team of clinicians aimed to reach a double goal of “safety and beauty” for the women included in this study. The data resulting from our study are only an initial step in showing that oncoplastic breast-conserving surgeries that perform restoration of body image are more appropriate in helping women with breast cancer to attain a higher quality of life. Although clinical elements of depression, anxiety, and stress were identified in both groups (without any statistically significant differences), we highlighted that feelings of desirability and femininity were much more prominent in women who underwent oncoplastic breast-conserving surgery when compared to women who experienced a modified radical mastectomy and delayed reconstruction. More interdisciplinary research focusing on oncological, surgical, imaging, anatomopathological, and psychiatric factors in women faced with a mastectomy, with and without immediate breast reconstruction, is essential for providing health professionals with the important and necessary coordinates for the most suitable case management.

## Figures and Tables

**Table 1:** Comparison of the socio-demographic data for the two groups of participants: Group I—patients who underwent oncoplastic breast-conserving surgery; Group II—patients who underwent a modified radical mastectomy and late breast reconstruction.

	Group I	Group II	*p*-Value
Number of participants	34	35	
Age [years] ^a^	49.0 ± 8.9	57.9 ± 7.1	<0.001 ^c^
Education			0.261 ^d^
High school	18 (52.9%)	19 (54.3%)	
Post-high-school–trade school	2 (5.9%)	6 (17.1%)	
University Graduate/Master’s/Ph.D.	14 (41.2%)	10 (28.6%)	
Marital status ^b^			0.305 ^d^
Married	24 (70.6%)	25 (71.4%)	
Unmarried	5 (14.7%)	4 (11.4%)	
Divorced	5 (14.7%)	3 (8.6%)	
Widower	0 (0%)	3 (8.6%)	
Location [urban] ^b^	25 (80.6%)	24 (66.7%)	0.198 ^d^

^a^ Continuous variables are indicated by their mean ± standard deviation; ^b^ categorical variables are presented by absolute frequency (percentage); ^c^ Independent samples *t* test (2-tailed); ^d^ Chi-square test (2-tailed).

**Table 2:** Comparison between the two groups (Group I—patients who underwent oncoplastic breast-conserving surgery; Group II—patients who underwent a modified radical mastectomy and late breast reconstruction) considering depression, anxiety, and stress levels, as described by the DASS-21 scale.

DASS-21 ^a^	Group I	Group II	*p*-Value
Depression ^b^			0.812 ^c^
Normal	10 (29.4%)	9 (25.7%)	
Mild–Moderate	12 (35.3%)	15 (42.9%)	
Severe–Extremely severe	12 (35.3%)	11 (31.4%)	
Anxiety ^b^			0.985 ^c^
Normal	8 (23.5%)	8 (22.8%)	
Mild–Moderate	11 (32.3%)	12 (34.3%)	
Severe–Extremely severe	15 (44.2%)	15 (42.9%)	
Stress ^b^			0.383 ^c^
Normal	20 (58.8%)	22(62.9%)	
Mild–Moderate	5 (14.7%)	8 (22.9%)	
Severe–Extremely severe	9 (26.4%)	5 (14.2%)	

^a^ DASS-21 contains 3 subscales, namely for Depression, Anxiety, and Stress, with each one including 7 items; the score for each subscale ranges from 0 to 21; ^b^ Data are presented as absolute frequency (percentage).; ^c^ Chi-square test (2-tailed).

**Table 3:** The group differences between the mean scores of the DASS 21 subscales.

DASS 21 Subscale	Group I (N = 34) ^a^	Group II (N = 35) ^a^	*p*-Value ^b^
Depression	9.84 ± 9.06	7.78 ± 4.77	0.204
Anxiety	9.65 ± 8.14	7.11 ± 4.67	0.117
Stress	8.32 ± 8.30	5.44 ± 4.25	0.074

^a^ Data are presented as mean ± standard deviation; ^b^ Student t test (2-tailed).

**Table 4:** Comparison between the two groups (Group I—patients who underwent oncoplastic breast-conserving surgery; Group II—patients who underwent a mastectomy and late breast reconstruction), according to the FACT-B test.

FACT-B ^a^	GROUP I (N = 34) ^b^	GROUP II (N = 35) ^b^	*p*-Value ^c^
Physical well-being	21.68 ± 5.07 (10–28)	22.33 ± 4.22 (13–28)	0.565
Social well-being	21.29 ± 5.24 (9–28)	19.56 ± 5.57 (7–28)	0.196
Psycho-emotional well-being	16.32 ± 2.59 (10–20)	14.89 ± 2.84 (7–20)	0.035
Functional well-being	19.45 ± 3.95 (12–27)	17.11 ± 3.67 (9–27)	0.001

^a^ The four subscales of FACT-B: physical, social, psycho-emotional and functional well-being; ^b^ Data presented as mean ± standard deviation (minimum-maximum); ^c^ Student t test (2-tailed).

**Table 5:** Comparison of the FACT-B B4 item (“I feel sexually attractive”) frequency between Groups I and II.

Item B4 Score	Group I (N = 34)	Group 2 (N = 35)	*p*-Value
Level 0	0 (0.0%)	7 (20.0%)	<0.001
Level 1	2 (5.8%)	14 (40.0%)	
Level 2	0 (0.0%)	14 (40.0%)	
Level 3	14 (41.1%)	0 (0.0%)	
Level 4	18 (52.9%)	0 (0.0%)	

**Table 6:** Comparison of the FACT-B B9 item (“I am able to feel like a woman”) frequency between Groups I and II.

Item B9 Score	Group I (N = 34)	Group 2 (N = 35)	*p*-Value
Level 0	0 (0.0%)	1 (2.9%)	0.001
Level 1	1 (3.0%)	3 (8.6%)	
Level 2	4 (11.7%)	16 (45.7%)	
Level 3	12 (35.3%)	11 (31.4%)	
Level 4	17 (50.0%)	4 (11.4%)	

## Data Availability

All data generated and/or analyzed during the present study are included in this published article.
